# Dental discoloration caused by Grey-MTAFlow cement: analysis of its physicochemical, biological and antimicrobial properties

**DOI:** 10.1590/1678-7757-2020-0269

**Published:** 2020-08-05

**Authors:** Lauter Eston PELEPENKO, Flávia SAAVEDRA, Gabriela Fernanda BOMBARDA, Brenda Paula Figueiredo de Almeida GOMES, Adriana DE-JESUS-SOARES, Alexandre Augusto ZAIA, Marco Antonio Hungaro DUARTE, Mario TANOMARU-FILHO, Marina Angélica MARCIANO

**Affiliations:** 1 Universidade de Campinas Faculdade de Odontologia de Piracicaba PiracicabaSão Paulo Brasil Universidade de Campinas, Faculdade de Odontologia de Piracicaba, Piracicaba, São Paulo, Brasil; 2 Universidade de São Paulo Faculdade de Odontologia de Bauru BauruSão Paulo Brasil Universidade de São Paulo, Faculdade de Odontologia de Bauru, Bauru, São Paulo, Brasil; 3 Universidade Estadual Paulista Faculdade de Odontologia de Araraquara AraraquaraSão Paulo Brasil Universidade Estadual Paulista (UNESP), Faculdade de Odontologia de Araraquara, Araraquara, São Paulo, Brasil.

**Keywords:** Tooth discoloration, Dental materials, Bismuth, Zinc oxide

## Abstract

Tricalcium silicate-based cement are materials used in reparative and regenerative procedures in endodontics. A recently proposed formulation aimed to enhance handling during clinical use with a versatile material applicable by syringe. Although, the use of bismuth oxide as radiopacifier and grey raw powder are drawbacks considering aesthetics.

**Objectives:**

Evaluate physicochemical, biological, and antimicrobial properties of Grey-MTAFlow (Ultradent) and assess whether the addition of zinc oxide (ZnO) prevents dentinal discoloration caused by bismuth oxide.

**Methodology:**

Grey-MTAFlow was manipulated in 'thin' consistency for all tests. Luminosity, color change, ion migration to dentine, radiopacity, setting time, ISO 6876:2012 linear flow, volumetric lateral flow and central filling of simulated grooves scanned using micro-computed tomography (μCT), pH, calcium release, volumetric change using μCT, chemical characterisation, cytotoxicity, and antimicrobial activity were assessed. Addition of 5% ZnO to Grey-MTAFlow and a bismuth-containing experimental composition were comparatively tested. Statistical analyses used Shapiro-Wilk, T-test, ANOVA, and Kolmogorov-Smirnov (p<0.05).

**Results:**

The addition of ZnO to Grey-MTAFlow prevented dentine darkening after 90 days due to bismuth migration reduction, although no statistical difference was found (p=0.863). ZnO addition significantly enhanced Grey-MTAFlow radiopacity without differences in initial setting time. Grey-MTAFlow presented an ISO linear flow of 10.9 mm and a balanced volumetric lateral flow with central filling in μCT evaluation. All compositions presented an alkaline pH after immersion. Grey-MTAFlow had a significantly higher calcium ion release after 28 days in comparison to 24 hours (p=0.011) and volumetric expansion of 0.4±1.8% after immersion. ZnO addition altered the hydrated cement matrix once calcium hydroxide (portlandite) could not be detected in characterisation. Neither of the materials produced inhibition halos nor reduced bacterial turbidity, but all presented cytocompatibility above 100%.

**Conclusion:**

Grey-MTAFlow expanded after immersion and exhibited higher luminosity values after the evaluation period when ZnO was added, but chemical modifications after this addition occurred.

## Introduction

Tricalcium silicate-based cement has been widely used for endodontic therapy since the 1990s, when mineral trioxide aggregate (MTA) was introduced.^1^ These types of cement are essential to the range of endodontic materials due to its repair-inductive biological properties.^2^ One of the shortcomings with the use of MTA is the susceptibility to tooth discoloration, which is caused by the interaction between the bismuth oxide contained in its formulation with dental hard structures^3^ and sodium hypochlorite used during root canal therapy.^4 , 5^

The radiopacifier bismuth oxide present in the MTA composition was shown to interact with dentine and other components, resulting in the migration of ions into the dentine and tooth staining.^3 , 4 , 6^ Bismuth oxide is beneficial regarding radiopacity due to its high molecular weight (465.96 g/mol), which requires small amounts for an ideal level of radiopacity (i.e., 3 mm of Al - ISO 6876:2012) compared to alternative radiopacifiers, such as calcium tungstate (287.92 g/mol), and zirconium oxide (123.21 g/mol).^7^

Grey-MTAFlow cement (Ultradent Products Inc., South Jordan, UT, USA) containing tricalcium and dicalcium silicate and bismuth oxide has recently been introduced in the market. This material is available in powder and water-based gel and can be prepared at different consistencies, namely: thin, thick, and putty. Its thin consistency allows it to be delivered with syringe and needle, which facilitates its insertion clinically. Grey-MTAFlow cement has an alkalinizing capability, low solubility, adequate radiopacity, biocompatibility, and induction of mineralisation.^8 , 9^ However, no studies have yet evaluated its color stability. This material is grey, and the presence of bismuth oxide in its composition is a factor that may cause discoloration after contact with the dentine.^3^

Aluminum fluoride^10^ and zinc oxide (ZnO)^11^ were added in small amounts to bismuth-containing formulations and tested in order to keep bismuth oxide as radiopacifier, but avoiding color changes. The addition of ZnO to MTA Angelus in small amounts (5%) prevented dental discoloration caused by bismuth oxide.^11^ The addition of ZnO to a different composition material such as Grey-MTAFlow cement might prevent an expected darkening of the dental structures.

This study aimed to investigate the physicochemical, biological, and antimicrobial properties of Grey-MTAFlow in comparison to the 5% ZnO in-weight added to this commercial formulation and an experimental cement composed of white tricalcium silicate, bismuth oxide and similar addition of 5% ZnO. The hypothesis tested is that Grey-MTAFlow cement containing bismuth oxide causes dental discoloration, which can be inhibited by adding 5% ZnO without significant properties alterations.

## Methodology

### Preparation of the materials

**Figure 1:**
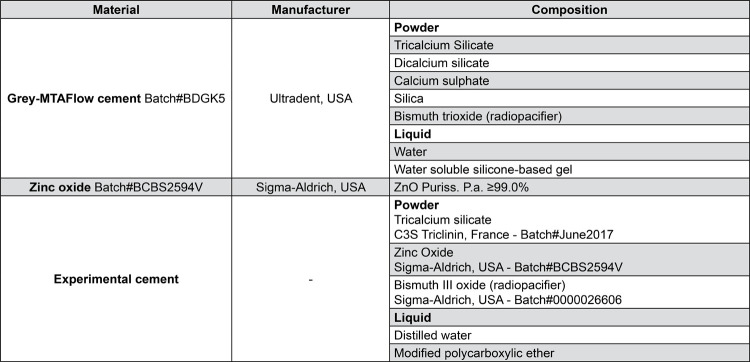
Composition and batch of the materials used in the analysis

The materials’ composition and batches are listed in Figure 1: 

Grey-MTAFlow cement (Ultradent, Ultradent Products Inc., South Jordan, UT, USA);

Grey-MTAFlow cement + 5% ZnO (Sigma-Aldrich, St. Louis, Missouri, USA);

Experimental cement proportioned using 80% tricalcium silicate (Mineral Research Processing, Meyzieu, France), 20% bismuth oxide (Sigma-Aldrich, St. Louis, Missouri, USA), and 5% ZnO (Sigma-Aldrich), added in weight. The liquid consisted of 95% distilled water and 5% modified polycarboxylic ether.

Both Grey-MTAFlow compositions were mixed in ‘thin’ consistency (1 big-end plus 1 small-end spoon [0.19 g] to 3 drops) following the manufacturers’ recommended powder to liquid ratio in order to obtain the best fluidity of this material. ZnO was added to the Grey-MTAFlow cement’s original formula in-weight at a percentage of 5% by using an electronic analytical scale (Gehaka AND-GR-202, Tokyo, Japan) with 10^-3^ precision. The experimental cement was mixed in a constant ratio of 1 g of powder to 0.25 mL of liquid.

### Color change in dentine

A total of 25 stain-free bovine teeth were selected for this study, according to a previously reported methodology^3^ . Samples were sectioned to obtain enamel-dentine blocks measuring 10×10 mm and thickness standardised in 3.5±0.1 mm. A cavity of 5.0 mm in diameter and 1.5 mm in depth was prepared at the centre of the dentine surface using a high-speed number 4054 bur (Medical Burs Sorensen, São Paulo, SP, Brazil). The specimens were then immersed in 1% sodium hypochlorite for 30 minutes, washed, immersed in 20% EDTA (pH 7.7) for 2 minutes, irrigated with a final flush of distilled water, and dried with gauze. Only the edges of the cavities were conditioned with 37% phosphoric acid for 30 seconds, and a layer of adhesive (Adper Single Bond 2, 3M ESPE, Sumaré, SP, Brazil) was applied only to the conditioned edge of the cavity and then light-cured (Optilight LD Max, Gnatus, Ribeirão Preto, SP, Brazil) for 20 seconds. In sequence, the tested compositions were inserted into the cavities at a depth of 1.5 mm. After the initial setting time, the cavities were sealed with a natural flow resin (B2, Nova DFL, Rio de Janeiro, RJ, Brazil). The polymerization was performed with a LED curing light unit (Optilight LD Max) for 60 seconds, and the specimens were stored in separate flasks containing tap water at 37^o^C throughout the test period. Triple antibiotic paste (metronidazole, ciprofloxacin, and minocycline), and unfilled samples served as positive and negative controls, respectively.

Luminosity (L) assessment was performed before filling, 24 hours, 28 days, and 90 days after filling. A spectrophotometer (Vita EasyShade V, VITA Zahnfabrik, Bad Sackingen, Germany) was used to obtain the values of the International Commission on Illumination (CIE) ‘L,’ ‘a’ and ‘b’ in a light-controlled room. The values were recorded and the color change (ΔE) between 90 days and before the filling was calculated by using the following formula: $∆E\;=\;{\left[{\left(L_{1}\;-\;L_{0}\right)}^{2}\;+\;{\left(a_{1}-a_{0}\right)}^{2}+{\left(b_{1}-b_{0}\right)}^{2}\right]}^{1/2}$ where ‘0’ stands for values before filling and ‘1’ the 90-days values.

After 90 days, representative specimens were sectioned, viewed under a stereomicroscope (SZX9, Olympus, Tokyo, Japan) at 2x magnification and images acquired using the software AxioVision (Carl Zeiss, Jena, Germany). In sequence, sectioned samples were resin-embedded, carbon-coated, and submitted to analysis under a scanning electron microscope (SEM) (JSM 5600 Lv JEOL, Akishima, Tokyo, Japan) and energy-dispersive spectroscopy (EDS) (JSM-5600/LvJEOL, Tokyo, Japan) regarding material/dentine interface ion migration.

### Radiopacity

Radiopacity was evaluated according to the ISO 6876:2012 standard and a previously established methodology^7^ . Three metallic rings *per* group were used to shape the cement specimens (10×1 mm), which were kept at 37±1°C and relative humidity until the final set. After this period, the specimens were radiographed with digital sensor (Micro Imagem, Indaiatuba, Sao Paulo, Brazil) at 60 kV, 10 mA, 0.3-second exposure, and focus-film distance of 30 cm. Instead of a radiographic film, a digital sensor was used to avoid film processing effects. An aluminum scale was used as a comparative radiographic density, and samples were evaluated in grey-scale values and converted into aluminum equivalent thickness (mm Al) using the Image J software (National Institutes of Health, Bethesda, MD, USA).

### Setting time

ISO 6876:2012 standard and a previously reported methodology^12^ were followed for setting time analysis. Freshly mixed cement was placed into metallic rings of 10 mm internal diameter and 2 mm thickness (n=3). The specimens were kept at a temperature of 37±1°C and humidity of 95±5% during the test and periodically subjected to vertical pressure by using Gilmore needles (according to ASTM-266/2008) in controlled room temperature at 20±1°C, in which a needle weighing 113.4 g was used for initial setting time and one weighing 453.6 g for final setting time. The setting time (in minutes) was determined from the mixing to the moment that it was no longer possible to observe the marking of each needle on the surface of the specimens.

### Flow analysis

For flow analysis, two different methodologies were used. First, according to the ISO 6876:2012 standard, a total volume of 0.05 mL of each cement (n=3) was placed at the centre of a flat smooth glass plate by using a plastic syringe. After 3 minutes from the initial mixing, a glass plate weighting 20±0.5 g was positioned above the cement dose along with another 100 g-weight. After 10 minutes, the mean between the highest and lowest diameters measures was considered as the cement flow (in mm).

The second flow test was performed as previously reported^13^ based on both linear and three-dimensional cement flow in simulated grooves scanned using micro-computed tomography (μCT) scans. Fifteen glass plates (n=5) were fabricated with a central cavity of 2 mm^3^ and four lateral grooves of 12 mm each. An amount of 0.05±0.005 mL of each freshly mixed material was placed into the central cavity. Next, a glass plate (20±0.5 g) and an extra weight (100 g) were immediately superposed for ten minutes. Linear lateral flow (in mm) was represented by the amount of material present in the lateral grooves, expressed by the average of the four measurements in the lateral grooves from the central cavity. For volumetric flow, both the lateral volumetric flow and central cavity filling and were evaluated. The specimens were scanned using a μCT scanner (SkyScan 1174, Bruker, Kontich, Belgium) with a 0.5-mm aluminum filter, 31.03-μm pixel size, ٣٦٠° rotation with 1.0-degree step, reconstructed with NRecon software (Bruker, Kontich, Belgium) and CTan software (Bruker, Kontich, Belgium) region of interest tool was used for analysis. The lateral volumetric flow was represented by the average material volume of the 2-mm far from the central cavity of the four lateral grooves (in mm^3^). Central cavity filling was obtained considering the volume of material present in this region (in mm^3^).

### pH and calcium ion release in solution

Fifteen acrylic teeth (n=5) with standardised root-end cavity with 3 x 1±0.1 mm (depth versus width) were used for assessment.^12^ The cavities were filled with the tested cement and immersed in individual flasks containing 10 mL of deionized water. Experimental periods of 3 hours, 24 hours and 28 days were used (this was due to the expected completion of the setting reaction of tricalcium silicate-based materials^14^ ). For analysis of pH, a calibrated pH-meter (371; Micronal, Sao Paulo, Brazil) was used, and measurements performed in each period. Standard calcium solutions were used as a reference for calcium ion release and evaluated by using atomic absorption spectroscopy (AA6800; Schimadzu, Tokyo, Japan) equipped with a calcium-specific hollow cathode lamp, the calcium ion release results were expressed in mg/L.

### Volume change

Volume change was evaluated by using a μCT scanner based on a material’s amount compatible with a surgical root-end cavity 3 × 1±0.1 mm as previously reported.^12^ Cavities were filled with the cement (n=5), and the initial scanning was performed according to the same previously described parameters. After scanning, each specimen was individually immersed in flasks containing 15 mL of deionized water and stored at 37ºC for 28 days. After this period, they were removed from the flasks, dried with filter paper, and re-scanned with the same initial parameters. The values of re-scanned volume were compared to the initial ones, representing the percentage volume change.

### Chemical characterisation

For chemical characterization,^15^ x-ray diffraction (XRD), scanning electron microscopy (SEM) and energy-dispersive X-ray spectroscopy (EDS) were used. Cement in un-hydrated and hydrated forms was evaluated using XRD. For analysis of un-hydrated cement, the powders were inserted directly into the sample holder of the XRD diffractometer. For hydrated cement, cylindrical specimens of 15×2 mm were prepared and allowed to set at 37^o^C and relative humidity of 95±5% for 24 hours, immersed in separate flasks containing 10 mL of Hank’s Balanced Salt Solution (HBSS) (Sigma-Aldrich Brazil Ltda, São Paulo, Brazil) and kept for 28 days at 37°C. The HBSS was changed weekly. After the immersion period, samples were dried, vacuum-desiccated, and crushed to a fine powder by using an agate mortar and pestle. The XRD diffractometer (Bruker D8 Advance, Bruker Corp., Billerica, MA, USA) was set at CuKa radiation, 40 mA, 45 kV, and adjusted to rotate between 10° - 60° with 0.02 degree, ٢θ step and step time of ٠.٦ seconds. Phase identification was undertaken by using search-match software (Diffrac.Eva, Bruker Corp., Billerica, MA, USA) under the International Centre for Diffraction Data (ICDD database, Newtown Square, PA, USA).

For SEM (Zeiss MERLIN Field Emission SEM, Carl Zeiss NTS GmbH, Oberkochen, Germany) and coupled EDS analysis, hydrated samples were epoxy resin-embedded, polished and carbon-coated. Images of the microstructural components of the different materials were obtained in back-scatter electron mode, and EDS in the selected area was carried out.

### Cytotoxicity - Methyl-thiazol-diphenyltetrazolium (MTT)

The cytotoxicity of the tested materials was assessed in human periodontal ligament fibroblasts harvested from the periodontal ligament of surgically removed third molars (B041 - Periocells of Periodontics Division of the Piracicaba Dental School, SP, Brazil [Ethics code: CAAE 20189119.7.0000.5418]). MTT assay was performed after 24 hours of contact between the materials and cells. Quintuplicates of each material was placed in contact with the fibroblasts culture according to a previous methodology.^16^ Absorbance reading was measured at 490 nm, and the cell viability percentage was calculated by dividing the absorbance values of experimental wells by those of negative control group wells. At least three independent experiments were carried out to ensure the reproducibility of the results.

### Antimicrobial activity

#### Agar diffusion test

This analysis was based on a previously described methodology^17^ . *Enterococcus faecalis* (ATCC 29212) and *Porphyromonas gingivalis* (ATCC 49417) bacterial strains were previously sub-cultured on appropriate medium plates and particular gaseous conditions. *E. faecalis* was sub-cultured on agar plates containing brain heart infusion (BHI) + 5% defibrinated sheep blood (Ebefarma, Araras, SP, Brazil) and incubated for 18 hours at 37°C under aerobic conditions. *P. gingivalis* was sub-cultured on fastidious anaerobe agar (FAA) + 5% defibrinated sheep blood and incubated for 48 hours in an anaerobic chamber (Don Whitley Scientific, Bradford, UK) with a controlled atmosphere containing 80% N_2_, 10% CO_2_ and 10% H_2_.

Three sterile stainless-steel cylinders (4.0 × 1.0 × 10 mm; inner diameter of 6 mm) filled with freshly-spatulated material were put in contact with the inoculated surface for each microorganism in separate plates. Due to the silicone components in the liquid of the Grey-MTAFlow cement, an additional metallic disc containing only its water-based gel was also tested. A metallic cylinder containing a sterile paper disc impregnated with 2% chlorhexidine gel was used as a negative control. Plates were kept at room temperature for 30 minutes inside a laminar flow-chamber allowing initial cement setting before return to the appropriate storage. Inhibition diameters of microbial growth were measured (in mm) and recorded after incubation periods of 24 hours and 7 days for *E. faecalis* and 48 hours and 5 days for *P. gingivalis* . Additionally, the growth viability test on the agar surface adjacent to the tested metallic discs and SEM analysis of the Grey-MTAFlow surface in contact with each microorganism was performed after the final experimental period.

#### Direct contact

Direct contact tested was based on a previously reported methodology.^17^ Bacterial suspension of *E. faecalis* in BHI broth was adjusted spectrophotometrically at 800 nm (Spectronic 20d+, Milton Roy, Houston, USA) to match the transmittance of 90 T (equivalent to 0.5 McFarland scale = 1.5 × 10^8^). Hydrated cement samples with 0.10 g were used after 24 hours of storage at 37ºC. The following sets were tested: tubes 1, 2 and 3 containing the bacterial inoculum + cement sample; tube 4 containing the bacterial inoculum + felt paper disc impregnated with of 2% chlorhexidine gel (antimicrobial control); tube 5 containing the bacterial inoculum only (bacterial growth control); and tube 6 as blank BHI solution for spectrophotometer calibration purposes. Turbidity measurements were performed after the tubes were vortexed for five seconds: immediately, 3 hours, 24 hours, and 48 hours after contact with the tested materials.

Parallelly to the spectrophotometer readings, growth viability was checked by separately dripping 10 μL broth aliquots of each tube on BHI plates stored at 37°C during each period for analysis after 24 hours. Cement dissolution was considered with three tubes containing similar 0.10g cement samples in BHI without bacterial inoculum. The values of dissolution turbidity obtained from these parallel tubes were subtracted from the expressed results.

## Statistical analysis

For statistical analysis, the software BioEstat 5.3 (Mamiraua, Tefe, Brazil) and SPSS Statistics 24 (IBM, Armonk, NY, USA) were used. Data were assessed regarding normal distribution by using the Shapiro-Wilk test. Significance level was set at 5% (p<0.05). The sample size was determined for each test based on the corresponding previous methodology to express a test power of at least 80%.

T-test and one-way ANOVA were used for normally distributed values (i.e., ΔE, radiopacity, ISO 6876 flow, volumetric flow, pH, calcium ion release, and volume change). In contrast, Kolmogorov-Smirnov tests were used for non-normally distributed values (i.e., L, setting time, cytotoxicity, and antimicrobial direct contact).

## Results

### Color change in dentine

**Figure 2:**
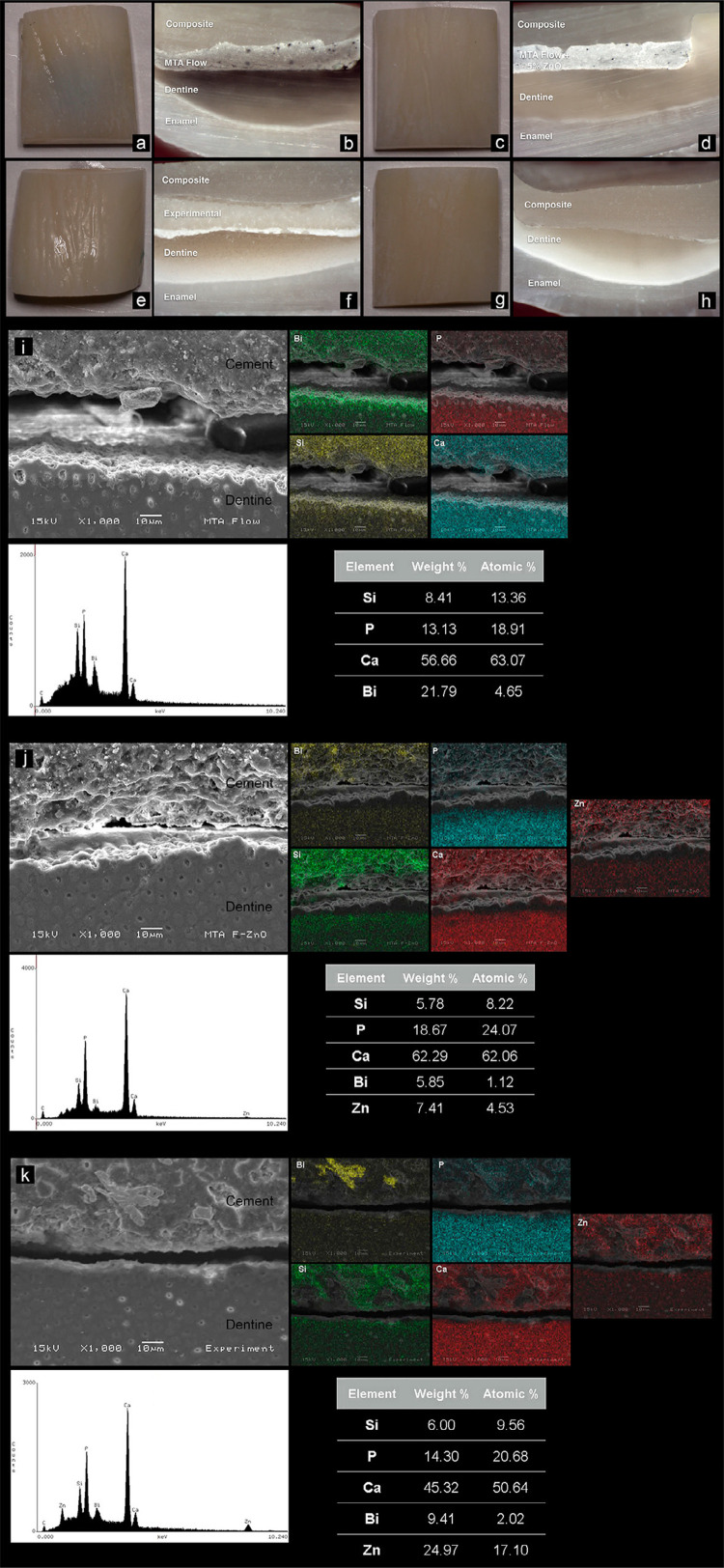
Representative specimens of bovine teeth filled with Grey-MTAFlow cement (a, b), Grey-MTAFlow cement + 5% ZnO (c, d), experimental cement (e, f) and negative control (g, h). Darkening is evident in Grey-MTAFlow cement. Dentine is stained, with grey color visible on the buccal surface. Stereomicroscopic images at 2x magnification. Scanning electron micrographs and energy dispersive analysis with elemental maps of sectioned teeth filled with the tested materials. Grey-MTAFlow cement (i), Grey-MTAFlow cement + 5% ZnO (j) and experimental cement (k). Calcium (Ca), silicon (Si), and phosphorus (P) were found in all specimens. Bismuth (Bi) was found in Grey-MTAFlow cement, Grey-MTAFlow cement + 5% ZnO and experimental cement corresponding to the radiopacifier. Zinc (Zn) was verified in Grey-MTAFlow cement + 5% ZnO and experimental cement corresponding to the additive. The migration of radiopacifier and Si into the dentine was evident in the elemental maps. The molecular weight of Bi was found to be high in the Grey-MTAFlow cement, with a high incidence of these ions at the cement/dentine interface. This was not verified in the Grey-MTAFlow cement + 5% ZnO, whose molecular weight of Bi was lower and ions evenly distributed in the material matrix.

Mean, standard deviation, and statistical difference for L and ΔE are shown in Table 1 . Tooth discoloration was evident in the buccal surface samples filled with Grey-MTAFlow ( Figure 2a ). Regarding L, Grey-MTAFlow presented the lowest mean value. Despite the visual color differences between Grey-MTAFlow and Grey-MTAFlow + 5% ZnO ( Figure 2b, d ), no statistical difference was found after 90 days (p=0.863). Stains in the dentine in Grey-MTAFlow samples were remarkable, indicating staining penetration inside the dentinal tubules. The addition of ZnO to Grey-MTAFlow resulted in a trend of increase in the luminosity after 90 days. For the experimental cement, L values were comparable to those of the negative control (p=0.826) after 90 days. A thin white layer could be observed between dentine and material matrix in the Grey-MTAFlow + 5% ZnO and experimental cement, probably due to ZnO precipitation during the setting time. Analysis of ΔE values showed that all tested materials exhibited color change after the experimental periods, with statistical differences with negative and positive controls (p<0.001).

**Table 1:** Mean and standard deviation values of color change (ΔE) after 90 days, and luminosity (L) at 24 hours, 28, and 90 days of analysis. Different lowercase letters in each column indicate statistically significant differences

Composition	Colour change	Luminosity		
	(ΔE)	24 hours	28 days	90 days
Negative control	6.3 ± 0.8^a^	92.4 ± 0.7^a^	90.0 ± 1.7^a^	90.7 ± 1.3^a^
Grey-MTAFlow cement	67.4 ± 1.7^bc^	85.1 ± 1.7^ab^	76.5 ± 0.5^abc^	67.8 ± 1.7^b^
Grey-MTAFlow + 5% ZnO	152.3 ± 6.2^c^	84.0 ± 0.4^bc^	73.1 ± 0.7^bcd^	74.2 ± 0.5^ab^
Experimental cement	48.5 ± 12.9^b^	94.0 ± 1.8^a^	78.3 ± 0.2^ad^	85.1 ± 4.0^a^
Positive control	1520.0 ± 28.4^d^	63.2 ± 13.0^bc^	44.5 ± 2.3^b^	30.1 ± 14.6^c^

SEM and elemental maps of the bovine teeth filled with the cement are shown in Figure 2 (i-k). The bismuth used as radiopacifier migrated from the materials into the tooth structure, being more intense at the dentine/material interface in the Grey-MTAFlow. Considering the Grey-MTAFlow + 5% ZnO interface, bismuth ions were more concentrated in the material matrix. The migration of Si into the dentine was also evident in the elemental maps for all tested cement.

### Radiopacity

The results for radiopacity are listed in Table 2 . All the tested compositions achieved a minimum of 3 mm of Al in a cement thickness of 1 mm as required by the ISO standard. A significant increase in radiopacity was observed for Grey-MTAFlow + 5% ZnO in comparison to pure Grey-MTAFlow (p=0.0004).

**Table 2:** Mean and standard deviation values of radiopacity, setting time, and flow. Different lowercase letters in each column indicate statistically significant differences between the tested materials

Composition	Radiopacity (mm Al)	Setting time (min)		Flow			
		Initial	Final	ISO 6876 (mm)	Linear lateral (mm)	Volumetric lateral (mm^3^) (max. 4 mm^3^)	Volumetric central cavity (mm^3^) (max. 2 mm^3^)
Grey-MTAFlow cement	4.94 ± 0.25^a^	8.12 ± 0.37^a^	49.00 ± 1.00^a^	10.90 ± 0.20^a^	6.70 ± 1.40^a^	2.40 ± 0.40^a^	1.10 ± 0.30^a^
Grey-MTAFlow + 5% ZnO	6.87 ± 0.16^b^	8.87 ± 0.12^a^	52.25 ± 0.25^b^	11.40 ± 0.20^b^	6.30 ± 0.60^a^	2.40 ± 0.20^a^	0.90 ± 0.20^a^
Experimental cement	6.00 ± 0.36^ab^	19.17 ± 0.76^b^	100.00 ± 2.00^c^	16.60 ± 0.60^b^	6.80 ± 0.90^a^	1.70 ± 0.40^a^	0.30 ± 0.20^b^

### Setting time

The results for the initial and final setting times are listed in Table 2 . The addition of ZnO did not significantly alter initial setting time of the Grey-MTAFlow (p=0.989). In opposition, ZnO increased the final setting time is approximately 3 minutes (p=0.022). Experimental cement showed significantly higher initial and final setting times in comparison to the other compositions (p<0.001).

### Flow analysis

The results for ISO 6876:2012 and volumetric flows are listed in Table 2 . ISO flow analysis showed that the experimental cement had a significantly higher flow, followed by Grey-MTAFlow + 5% ZnO, with statistical difference compared to the other materials (p<0.050). The volumetric analysis revealed that the experimental cement presented the lowest central cavity filling and lateral volumetric flow, probably due to its consistency (p<0.050). On the other hand, Grey-MTAFlow showed the highest volumetric filling in the central cavity, and ZnO addition did not alter the volumetric material filling (p>0.050).

### pH and calcium ion release in solution

The mean values of pH and calcium ion release obtained are shown in Table 3 . All the tested compositions presented alkaline pH ranging between 8 and 9. ZnO addition significantly increased the pH values of Grey-MTAFlow (p=0.022) after 28 days. No differences for pH values were observed between 24-hour and 28-day periods, regardless of the material (p=0.461).

**Table 3:** Mean and standard deviation values of pH, calcium ion release, volume change, and cytotoxicity. Different letters in each column indicate statistically significant differences between tested materials (lowercase) and periods (uppercase)

Composition	pH			Calcium ion release (mg/L)			Volume change*	Cytotoxicity
	3 hoursA	24 hoursA	28 daysA	3 hoursA	24 hoursA	28 daysB	(% after 28 days)	(%)
Grey-MTAFlow cement	8.06 ± 0.14^a^	8.02 ± 0.09^a^	8.07 ± 0.26^a^	4.77 ± 0.76^a^	4.35 ± 0.89^a^	12.35 ± 1.76^a^	+ 0.40 ± 1.80^a^	106.94 ± 13.24^a^
Grey-MTAFlow + 5% ZnO	8.12 ± 0.11^a^	8.14 ± 0.07^b^	8.20 ± 0.20^a^	1.50 ± 0.39^b^	5.78 ± 0.98^b^	9.80 ± 1.09^a^	- 0.50 ± 2.10^a^	109.00 ± 10.37^a^
Experimental cement	7.82 ± 0.17^b^	8.28 ± 0.10^c^	8.60 ± 0.37^b^	3.93 ± 0.70^a^	3.87 ± 0.54^a^	27.24 ± 3.18^b^	- 4.60 ± 1.30^b^	133.75 ± 12.71^a^

*Volume change negative values represent volume loss and positive values represents expansion.

Considering the calcium ion release, Grey-MTAFlow showed significantly higher ion release at 3-hours compared to Grey-MTAFlow + 5% ZnO (p<0.0001), but similar to that of the experimental cement (p=0.112). On the other hand, ZnO addition significantly increased the calcium ion release at the 24-hours period (p=0.043), although no difference was observed between these two compositions at 28 days (p=0.954). Considering the experimental periods, the 28-day period showed significantly higher values regardless of the material analysed (p=0.011).

### Volume change

The results for volume change are listed in Table 3 . Grey-MTAFlow presented a slight increase in volume (expressed as positive value) without significant difference compared to Grey-MTAFlow + 5% ZnO (p=0.947). Experimental cement had the highest percentage of volume reduction (i.e., 4.6%), being significantly more soluble than the other two compositions (p=0.003).

### Chemical characterization

**Figure 3:**
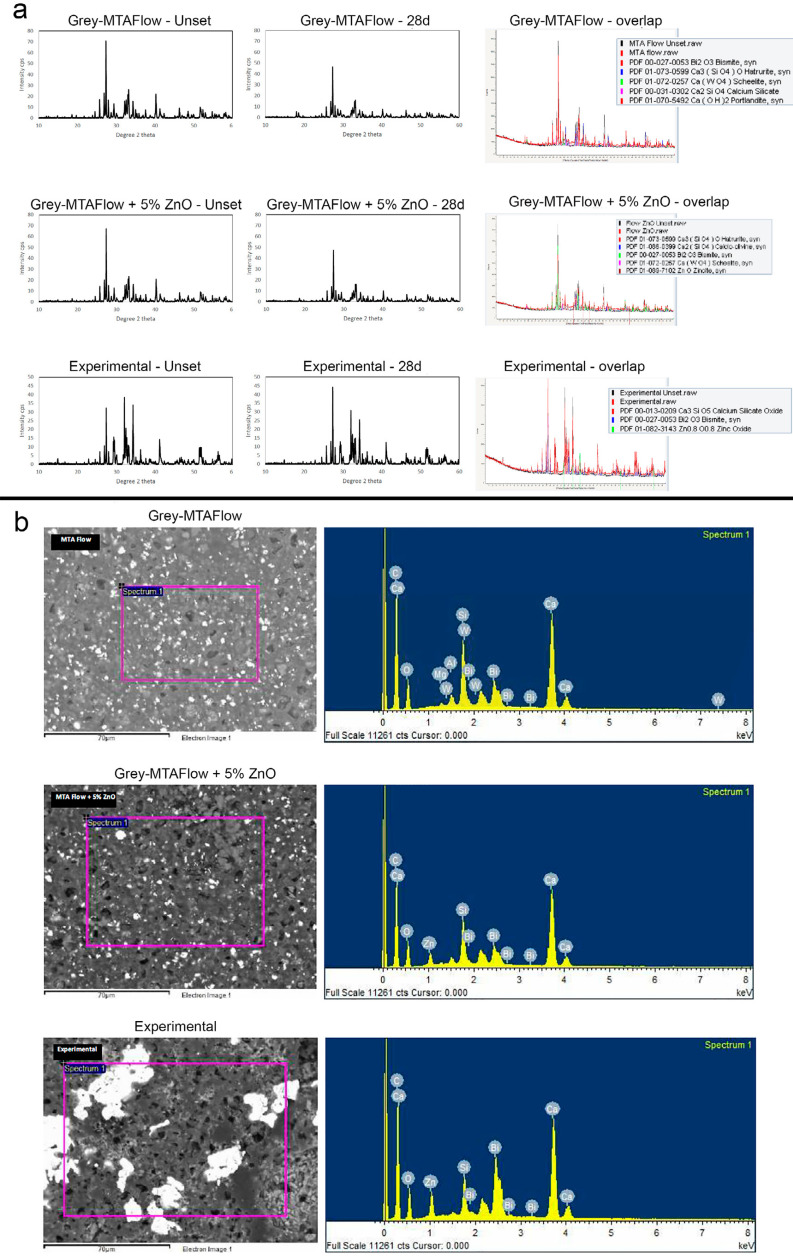
(a) X-ray diffraction plots of un-hydrated and hydrated cement and overlap diffraction. Calcium hydroxide (portlandite) was detected in hydrated Grey-MTAFlow cement and not observed in the other tested compositions. Scanning electron micrographs (SEM) and energy dispersive spectroscopy (EDS). (b) SEM micrographs and EDS plots of hydrated cement revealed particles of cement interposed by small particles of radiopacifier, with peaks detected. Experimental cement showed large particles of bismuth oxide, suggesting aggregation of this component

X-ray diffraction plots of the un-hydrated and hydrated cement and the diffraction overlap of them are shown in Figure 3a . Grey-MTAFlow presented peaks of tricalcium and dicalcium silicate (ICDD: 01-073-0599 and 00-031-0302, respectively) as well as of radiopacifier bismuth oxide (ICDD: 00-027-0053) and portlandite after the 28-day immersion in HBSS. Similar diffraction was detected for Grey-MTAFlow + 5% ZnO, except for peaks of ZnO detected (ICDD: 01-089-7102) due to the addition. For both Grey-MTAFlow and Grey-MTAFlow + 5% ZnO, a peak of calcium tungstate (scheelite) was verified (ICDD: 01-072-0257 and 01-089-7102, respectively). Scheelite is not listed in the composition of the Grey-MTAFlow stated by the manufacturer. Experimental cement was composed of tricalcium silicate (ICDD: 00-013-0209), bismuth oxide (ICDD: 00-027-0053), and ZnO (ICDD: 01-082-3143). ZnO addition altered the hydrated cement matrix once calcium hydroxide (portlandite) could not be detected in characterisation for these materials where ZnO was present.

Scanning electron micrographs and elemental analysis are represented in Figure 3b . SEM micrographs and EDS plots of hydrated cement revealed particles of cement interposed by small particles of radiopacifier, with peaks also detected. Experimental cement showed large particles of bismuth oxide, suggesting the aggregation of this component.

### Cytotoxicity

The viability of periodontal ligament fibroblasts in the presence of the tested compositions after 24 hours are represented in Table 3 . Cell viability levels were similar, and all tested compositions presented no cytotoxicity to periodontal ligament fibroblasts with values above 100%. Statistical analysis showed no significant difference between the materials comparatively to the negative control: Grey-MTAFlow (p=0.997), Grey-MTAFlow + 5% ZnO (p=0.994) and experimental cement (p=0.977).

### Antimicrobial activity

**Figure 4:**
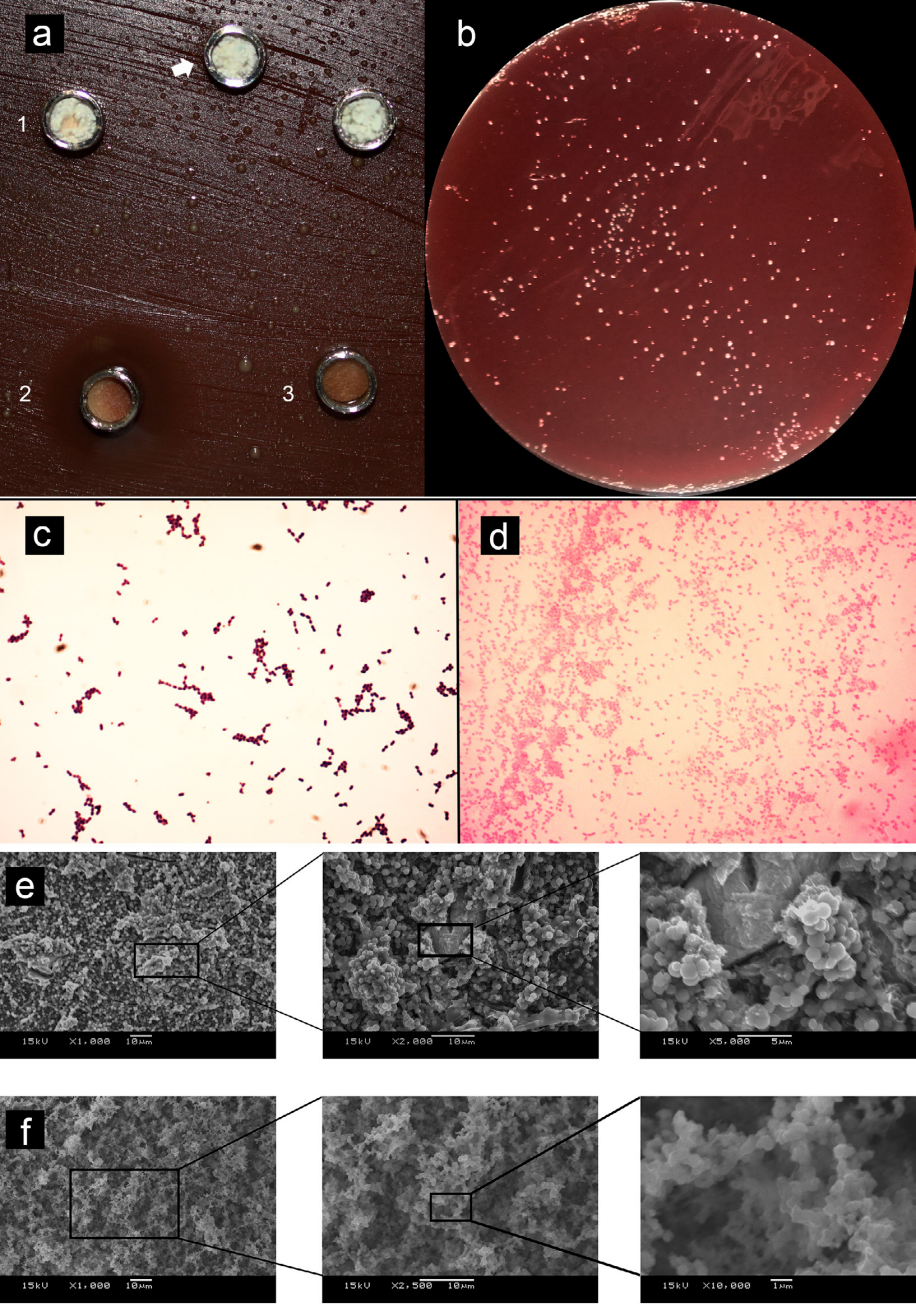
(a) - 1) Grey-MTAFlow cement without inhibition halos in BHI medium containing inoculated E. faecalis; (2) Chlorhexidine gel used as a control for antimicrobial activity presenting inhibition halo; (3) Additional metallic disc containing only silicon-based gel without inhibition halo as well; the arrow is indicating the collection area for smear and viability tests. (b) Viability test for E. faecalis after 7 days showing viable bacteria adjacent to the disc. (c) The smear of E. faecalis after 7 days in contact with fresh Grey-MTAFlow cement. (d) The smear of P. gingivalis after 5 days in contact with fresh Grey-MTAFlow cement. (e) Representative SEM images of the surface of Grey-MTAFlow in contact with E. faecalis at 1,000x, 2,000x, and 5,000x magnifications. (f) Representative SEM images of the surface of Grey-MTAFlow in contact with P. gingivalis at 1,000x, 2,500x, and 10,000x magnifications

No inhibition halos were observed for the cement, nor for the water-based gel tested, regardless of the bacteria ( Figure 4a ). Control presented halos with average diameters of 19.1 mm against *E. faecalis* and 42.2 mm against *P. gingivalis* , with significant difference (p<0.001) between the microorganisms. Bacterial growth viability was observed after 7 days of contact with the cement for *E. faecalis* ( Figure 4b ). The purity of cultures through Gram-staining was confirmed after the experimental periods ( Figure 4c, d ). SEM analysis of the cement’s surface in contact with bacteria showed biofilm structures on their surface, with detailed images at higher magnifications revealing bacterial presence ( Figure 4e, f ).

**Figure 5:**
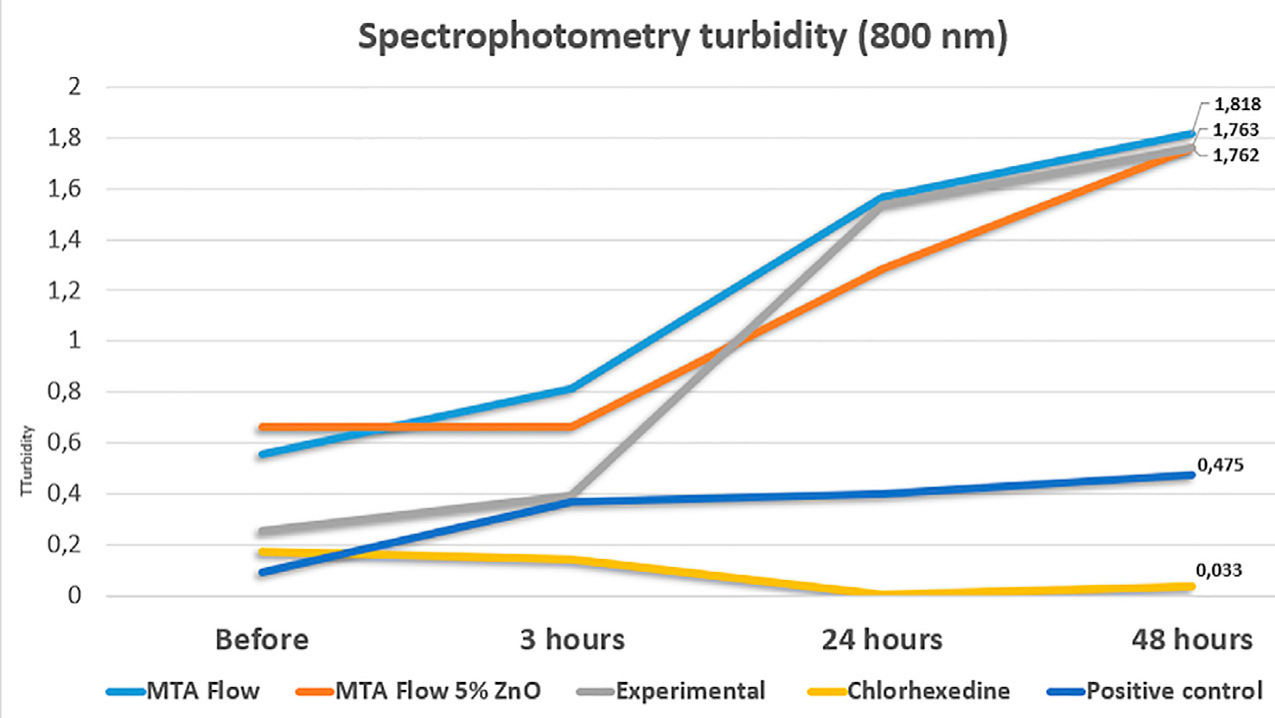
Spectrophotometric representation of the tested compositions presenting significantly higher values of turbidity (p<0.010) after discounting the solubility in comparison to positive control and chlorhexidine. Chlorhexidine presented the lowest values of turbidity after 3 hours (p=0.001)

Concerning the direct contact method, all cement presented significantly higher levels of turbidity after discounting their solubility when compared to both controls (p<0.010) ( Figure 5 ). Viable bacteria were re-collected from the tested cement and positive control in all experimental periods up to 48 hours. Chlorhexidine used as an antimicrobial control presented significantly lower values of turbidity after 3 hours (p=0.001) and presented no viable microorganisms up to 48 hours.

## Discussion

Tooth discoloration caused by MTA has been widely demonstrated both *in vitro* and clinically. This material drawback occurs due to the interaction between the radiopacifier bismuth oxide and dental structures.^3 , 18 , 19^ Bismuth oxide is present in the composition of the Grey-MTAFlow and, consequently, is expected a color change when in contact with dentine. A previous investigation^11^ showed that 5% ZnO used as an additive in a white-powder material (MTA Angelus) inhibited the destabilisation of bismuth oxide and consequent discoloration of the tooth. The present study tested the hypothesis that the addition of 5% ZnO into a grey-powder composition would also prevent tooth discoloration caused by this material. This hypothesis was partially accepted once L values after 90 days were higher after addition, although no significant difference was observed.

Aesthetics is an important aspect to be observed after dental restorative procedures.^20^ Tooth discoloration was evident in Grey-MTAFlow cement after 90 days as the staining of dentine was intense, where bismuth ions were detected. This finding is in accordance with previous studies,^3 , 5^ which found bismuth and silicon ions in dentine. The L values continually decreased after the insertion of Grey-MTAFlow, indicating a tendency of light absorbance. The addition of 5% ZnO to Grey-MTAFlow inhibited tooth discoloration visually, but the difference was not statistically significant compared to the group using pure Grey-MTAFlow. This fact can be attributed to the period of analysis that had a maximum of 90 days. The tendency of darkening in the group using pure Grey-MTAFlow would probably continue over time, and thus, a statistical difference could be detected. Regarding the experimental cement, high values of L were observed for this cement, suggesting that its color was stable and that ZnO prevented discoloration. In the sectioned specimens, there was an evident area of different color at the dentine/material interface.

The elemental map did not reveal a different composition for this layer, although probably the ZnO precipitated during the material setting time, which was longer than in other cement. Besides, a previous study^3^ stated that ZnO molecules interact with the bismuth oxide, stabilizing it from phase changes when in the presence of strong oxidizing agents. This observation could explain the color results obtained in the present study when ZnO was added. The addition of calcium chloride^21^ to the experimental cement formulation could reduce the setting time, preventing the precipitation of the components. The use of bovine teeth in the present study aimed to provide a sufficiently flat surface, enabling color assessments, and thus standardized measurements. Previous investigations did not report differences in dental discoloration pattern when using bovine or human teeth models.^3 , 5^

Radiopacity is a vital material characteristic and should be sufficient to allow distinction from dentin and adjacent anatomical structures in follow-up radiographic and tomographic exams.^7^ The results obtained in the present study showed that Grey-MTAFlow radiopacity was significantly enhanced by ZnO addition. This result could be explained by the additional radiopacity provided by the ZnO addition. However, all the tested compositions presented adequate radiopacity (i.e., at least 3 mm of Al), according to the ISO standard. A previous study^8^ found similar values of radiopacity for the Grey-MTAFlow.

Considering clinical use, facilitated handling^22^ is an essential characteristic for the material during its working time^23^ in order to provide an optimal insertion into cavities.^24^ Grey-MTAFlow cement presented proper handling and flow, probably due to the water-gel based liquid used for its hydration. The ISO flow test was initially designed for endodontic sealers and considered linear measurements only, but the clinical conditions in which the tested materials are inserted are three-dimensional. Tanomaru-Filho, et al.^13^ (2017) proposed a µCT evaluation of the flow for reparative cement considering its volumetric variations in opposition to the ISO tests. With this methodology, one can assess using simulated grooves the central filling and lateral flow. Grey-MTAFlow and Grey-MTAFlow + 5% ZnO presented similar volumetric values suggesting that the ZnO addition does not interfere with the volumetric flow. The experimental cement presented the lowest central filling, probably due to its consistency and significant higher setting times. No past studies have evaluated the volumetric filling of Grey-MTAFlow for comparison with the results obtained in the present study.

Considering the clinical procedures in which these reparative materials are used, short initial setting time is a critical characteristic.^8 , 25^ ZnO addition slightly prolonged the final setting time compared to that of pure Grey-MTAFlow. The experimental cement had the longest initial and final setting times, which is probably related to the particle size and prolonged hydration reaction as a result. Setting time is directly related to solubility, which was significantly higher in the experimental cement. Grey-MTAFlow and Grey-MTAFlow + 5% ZnO cement showed similar volume changes. However, Grey-MTAFlow cement presented a slight volume increase after immersion, probably due to water absorption during the immersion period. This increase in volume was previously reported in conventional MTA formulations.^25^ Volume loss (1.2%) was previously reported^8^ for Grey-MTAFlow cement when tested with a similar methodology using the ‘putty’ consistency. The ‘thin’ consistency used in the present study might be the reason for this slight variability in this result mainly.

An alkaline environment caused by hydroxyl release and precipitation of calcium hydroxide is crucial for repair.^26^ Previous studies reported higher values of alkaline pH as a result of hydration and calcium ion release in solution.^27^ In the present study, all the tested materials presented pH values of above 8 after a 28-day immersion in water. Guimarães, et al.^8^ (2017) found higher pH values for Grey-MTAFlow cement, whose ‘putty’ consistency was possibly associated with higher solubility and calcium ion release in water. For the experimental cement, calcium release was significantly higher after 28 days, suggesting that a higher volume loss contributed to these increased values. A balance between material-matrix compounds release and stability is vital to provide sealing after long periods since these materials are not expected to be replaced.

The material characterisation was performed for both un-hydrated and hydrated cement after 28 days of immersion in HBSS. This methodology is well documented in the literature and has been used to evaluate the composition of endodontic materials.^27 - 29^ In the present study, calcium hydroxide was detected in Grey-MTAFlow, but not in Grey-MTAFlow + 5% ZnO, and experimental cement. These results suggest that the presence of Zn affected calcium hydroxide crystalline phase deposition after hydration and, therefore, XRD analysis would not detect this component. Calcium hydroxide absence after hydration is the main shortcoming of the ZnO addition, once this precipitation would stimulate repair. Further biological studies using an animal model might elucidate if calcium hydroxide reduction affects tissue repair and mineralization ability. A similar phenomenon was previously described when ZnO was added to MTA Angelus, with calcium hydroxide not being detectable in XRD scans.^11^ For all cement, the phases detected were mainly tricalcium silicate and radiopacifier bismuth oxide (ICDD: 00-027-0053). Moreover, the radiopacifier compound calcium tungstate (scheelite) was detected in Grey-MTAFlow and Grey-MTAFlow + 5% ZnO (ICDD: 01-072-0257). According to the manufacturer, this component is not present in the cement formula, but the results from the present study suggest the contrary. Other characterisation methodologies could confirm the presence of this component in the Grey-MTAFlow composition.

Reparative materials remain in close contact with connective tissue, and for that reason, material cytocompatibility is an important feature.^22^ *In vitro* cytotoxicity assays are well-established methods for primary analyses of biological properties of reparative materials.^16^ All the tested materials had similar cytotoxicity levels of over 100% in comparison to the negative control. Thus, there are indications that the tested materials were non-cytotoxic. Further biological investigations considering cell proliferation, and live/dead quantification would confirm the cytocompatibility of these materials, once MTT does not provide live/dead cell quantification. A previously reported study^9^ showed proper biocompatibility and biomineralization induction for the Grey-MTAFlow after 7 to 60-days implantation in rats.

The antibacterial effect is expected from reparative endodontic materials.^30^ Two distinct methodologies were used to evaluate the antimicrobial activity of the cement: agar diffusion and direct contact methods. The results showed limited materials’ ability to inhibit bacterial growth, regardless of the methodology used or bacterial species tested. Similar results were reported in previous studies.^30 , 31^ SEM analysis of the cement’ surface left in contact with inoculated surfaces showed that all the materials presented biofilm on their surface. None of the previous studies performed SEM analysis of the cement discs after contact with bacteria. Misinterpretation of the halos can occur as a result of cement dissolution.^32^ This effect is an indication that the use of inhibition halos tests for hydraulic cement seems to be inadequate due to possible misleading interpretations. Considering the direct contact test, all materials presented similar turbidity values after discounted its solubility and viable bacteria for up to 48 hours. An apparent up-regulation of the bacterial growth occurred probably due to the calcium ion release in BHI that stimulated bacterial growth. Further studies with more extended analysis periods, considering pH evaluation, different bacterial species, and live/dead quantification, are necessary to clarify these findings.

## Conclusion

Grey-MTAFlow containing bismuth oxide as a radiopacifier can potentially cause dentine discoloration, which was influenced by the addition of ZnO considering luminosity. Physical properties were not affected by such addition, although chemical modifications were verified with no detection of calcium hydroxide deposition. Biologically, all the tested materials presented cytocompatibility, but no detectable antimicrobial activity. Besides, the use of an inert alternative radiopacifier would be of great advantage considering the long-term aesthetic outcome.

## References

[1] - Torabinejad M, Hong CU, McDonald F, Pitt Ford TR. Physical and chemical properties of a new root-end filling material. J Endod. 1995;21(7):349-53. doi: 10.1016/S0099-2399(06)80967-2 10.1016/S0099-2399(06)80967-2 7499973

[2] - Duarte MA, Marciano MA, Vivan RR, Tanomaru M Filho, Tanomaru JM, Camilleri J. Tricalcium silicate-based cements: properties and modifications. Braz Oral Res. 2018;32(suppl 1):111-8. doi:10.1590/1807-3107bor-2018.vol32.0070 10.1590/1807-3107bor-2018.vol32.0070 30365611

[3] - Marciano MA, Costa RM, Camilleri J, Mondelli RF, Guimarães BM, Duarte MA. Assessment of color stability of white mineral trioxide aggregate angelus and bismuth oxide in contact with tooth structure. J Endod. 2014;40(8):1235-40. doi: 10.1016/j.joen.2014.01.044 10.1016/j.joen.2014.01.044 25069940

[4] - Camilleri J. Color stability of white mineral trioxide aggregate in contact with hypochlorite solution. J Endod. 2014;40(3):436-40. doi: 10.1016/j.joen.2013.09.040 10.1016/j.joen.2013.09.040 24565667

[5] - Marciano MA, Duarte MA, Camilleri J. Dental discoloration caused by bismuth oxide in MTA in the presence of sodium hypochlorite. Clin Oral Investig. 2015;19(9):2201-9. doi: 10.1007/s00784-015-1466-8 10.1007/s00784-015-1466-8 25922130

[6] - Felman D, Parashos P. Coronal tooth discoloration and white mineral trioxide aggregate. J Endod. 2013;39(4):484-7. doi: 10.1016/j.joen.2012.11.053 10.1016/j.joen.2012.11.053 23522541

[7] - Duarte MA, Kadre GD, Vivan RR, Tanomaru JM, Tanomaru M Filho, Moraes IG. Radiopacity of portland cement associated with different radiopacifying agents. J Endod. 2009;35(5):737-40. doi: 10.1016/j.joen.2009.02.006 10.1016/j.joen.2009.02.006 19410095

[8] - Guimarães BM, Vivan RR, Piazza B, Alcalde MP, Bramante CM, Duarte MA. Chemical-physical properties and apatite-forming ability of mineral trioxide aggregate flow. J Endod. 2017;43(10):1692-6. doi: 10.1016/j.joen.2017.05.005 10.1016/j.joen.2017.05.005 28735787

[9] - Bueno CR, Vasques AM, Cury MT, Sivieri-Araújo G, Jacinto RC, Gomes-Filho JR, et al. Biocompatibility and biomineralization assessment of mineral trioxide aggregate flow. Clin Oral Investig. 2019;23(1):169-77. doi: 10.1007/s00784-018-2423-0 10.1007/s00784-018-2423-0 29572687

[10] - Marciano MA, Camilleri J, Lucateli RL, Costa RM, Matsumoto MA, Duarte MA. Physical, chemical, and biological properties of white MTA with additions of AlF3. Clin Oral Investig. 2019;23(1):33-41. doi: 10.1007/s00784-018-2383-4 10.1007/s00784-018-2383-4 29654562

[11] - Marciano MA, Camilleri J, Costa RM, Matsumoto MA, Guimarães BM, Duarte MA. Zinc oxide inhibits dental discoloration caused by white mineral trioxide aggregate Angelus. J Endod. 2017;43(6):1001-7. doi: 10.1016/j.joen.2017.01.029 10.1016/j.joen.2017.01.029 28416317

[12] - Cavenago BC, Pereira TC, Duarte MA, Ordinola-Zapata R, Marciano MA, Bramante CM, et al. Influence of powder-to-water ratio on radiopacity, setting time, pH, calcium ion release and a micro-CT volumetric solubility of white mineral trioxide aggregate. Int Endod J. 2014;47(2):120-6. doi: 10.1111/iej.12120 10.1111/iej.12120 23647286

[13] - Tanomaru-Filho M, Torres FF, Bosso-Martelo R, Chávez-Andrade GM, Bonetti-Filho I, Guerreiro-Tanomaru JM. A novel model for evaluating the flow of endodontic materials using micro–computed tomography. J Endod. 2017;43(5):796-800. doi: 10.1016/j.joen.2016.12.002 10.1016/j.joen.2016.12.002 28268019

[14] - Camilleri J, Montesin FE, Papaioannou S, McDonald F, Pitt Ford TR. Biocompatibility of two commercial forms of mineral trioxide aggregate. Int Endod J. 2004;37(10):699-704. doi: 10.1111/j.1365-2591.2004.00859.x 10.1111/j.1365-2591.2004.00859.x 15347295

[15] - Marciano MA, Duarte MA, Camilleri J. Calcium silicate-based sealers: assessment of physicochemical properties, porosity and hydration. Dent Mater. 2016;32(2):e30-e40. doi: 10.1016/j.dental.2015.11.008 10.1016/j.dental.2015.11.008 26727694

[16] - Silva EJ, Senna PM, De-Deus G, Zaia AA. Cytocompatibility of Biodentine using a three-dimensional cell culture model. Int Endod J. 2016;49(6):574-80. doi: 10.1111/iej.12485 10.1111/iej.12485 26100656

[17] 17 - Gomes BP, Pedroso JA, Jacinto RC, Vianna ME, Ferraz CC, Zaia AA, al. *In vitro* evaluation of the antimicrobial activity of five root canal sealers. Braz Dent J. 2004;15(1):30-5. doi: 10.1590/S0103-64402004000100006 10.1590/s0103-64402004000100006 15322642

[18] - Belobrov I, Parashos P. Treatment of tooth discoloration after the use of white mineral trioxide aggregate. J Endod. 2011;37(7):1017-20. doi: 10.1016/j.joen.2011.04.003 10.1016/j.joen.2011.04.003 21689563

[19] - Ioannidis K, Mistakidis I, Beltes P, Karagiannis V. Spectrophotometric analysis of coronal discoloration induced by grey and white MTA. Int Endod J. 2013;46(2):137-44. doi: 10.1111/j.1365-2591.2012.02098.x 10.1111/j.1365-2591.2012.02098.x 22823058

[20] - Davis LG, Ashworth PD, Spriggs LS. Psychological effects of aesthetic dental treatment. J Dent. 1998;26(7):547-54. doi: 10.1016/s0300-5712(97)00031-6 10.1016/s0300-5712(97)00031-6 9754742

[21] - Bortoluzzi EA, Broon NJ, Bramante CM, Felippe WT, Tanomaru-Filho M, Esberard RM. The influence of calcium chloride on the setting time, solubility, disintegration, and pH of mineral trioxide aggregate and white Portland cement with a radiopacifier. J Endod. 2009;35(4):550-4. doi:10.1016/j.joen.2008.12.018 10.1016/j.joen.2008.12.018 19345803

[22] - Parirokh M, Torabinejad M. Mineral trioxide aggregate: a comprehensive literature review-part lll: clinical applications, drawbacks, and mechanism of action. J Endod. 2010;36(3):400-13. doi: 10.1016/j.joen.2009.09.009 10.1016/j.joen.2009.09.009 20171353

[23] - Nowicka A, Lipski M, Parafiniuk M, Sporniak-Tutak K, Lichota D, Kosierkiewicz A, et al. Response of human dental pulp capped with biodentine and mineral trioxide aggregate. J Endod. 2013;39(6):743-7. doi: 10.1016/j.joen.2013.01.005 10.1016/j.joen.2013.01.005 23683272

[24] - Alcalde MP, Vivan RR, Marciano MA, Duque JA, Fernandes SL, Rosseto MB, et al. Effect of ultrasonic agitation on push-out bond strength and adaptation of root-end filling materials. Restor Dent Endod. 2018;43(2):e23. doi: 10.5395/rde.2018.43.e23 10.5395/rde.2018.43.e23 PMC5952061 29765903

[25] - Duque JA, Fernandes SL, Bubola JP, Duarte MA, Camilleri J, Marciano MA. The effect of mixing method on tricalcium silicate-based cement. Int Endod J. 2018;51(1):69-78. doi: 10.1111/iej.12774 10.1111/iej.12774 28370026

[26] - Gandolfi MG, Siboni F, Botero T, Bossù M, Riccitiello F, Prati C. Calcium silicate and calcium hydroxide materials for pulp capping: biointeractivity, porosity, solubility and bioactivity of current formulations. J Appl Biomater Funct Mater. 2015;13(1):43-60. doi: 10.5301/jabfm.5000201 10.5301/jabfm.5000201 25199071

[27] - Camilleri J. Evaluation of the effect of intrinsic material properties and ambient conditions on the dimensional stability of white mineral trioxide aggregate and Portland cement. J Endod. 2011;37(2):239-45. doi: 10.1016/j.joen.2010.11.012 10.1016/j.joen.2010.11.012 21238810

[28] - Camilleri J. Hydration mechanisms of mineral trioxide aggregate. Int Endod J. 2007;40(6):462-70. doi: 10.1111/j.1365-2591.2007.01248.x 10.1111/j.1365-2591.2007.01248.x 17459120

[29] - Camilleri J. Characterization of hydration products of mineral trioxide aggregate. Int Endod J. 2008;41(5):408-17. doi: 10.1111/j.1365-2591.2007.01370.x 10.1111/j.1365-2591.2007.01370.x 18298574

[30] - Farrugia C, Baca P, Camilleri J, Arias Moliz MT. Antimicrobial activity of ProRoot MTA in contact with blood. Sci Rep. 2017;7:41359. doi: 10.1038/srep41359 10.1038/srep41359 PMC5269669 28128328

[31] - Jardine AP, Montagner F, Quintana RM, Zaccara IM, Kopper PM. Antimicrobial effect of bioceramic cements on multispecies microcosm biofilm: a confocal laser microscopy study. Clin Oral Investig. 2019;23(3):1367-72. doi: 10.1007/s00784-018-2551-6 10.1007/s00784-018-2551-6 30022269

[32] - Farrugia C, Haider J, Camilleri L, Camilleri J. Clinical relevance of antimicrobial testing results for dental restorative materials. J Appl Biomater Funct Mater. 2017;15(2):e153-e61. doi: 10.5301/jabfm.5000337 10.5301/jabfm.5000337 28256700

